# Multiscale Attention Fusion for Depth Map Super-Resolution Generative Adversarial Networks

**DOI:** 10.3390/e25060836

**Published:** 2023-05-23

**Authors:** Dan Xu, Xiaopeng Fan, Wen Gao

**Affiliations:** 1 School of Computer Science and Technology, Harbin Institute of Technology, Harbin 150001, China; 2 Pengcheng Laboratory, Shenzhen 518052, China; 3 School of Electronic Engineering and Computer Science, Peking University, Beijing 100871, China

**Keywords:** attention, depth map, fusion, generative adversarial networks, multiscale, super-resolution

## Abstract

Color images have long been used as an important supplementary information to guide the super-resolution of depth maps. However, how to quantitatively measure the guiding effect of color images on depth maps has always been a neglected issue. To solve this problem, inspired by the recent excellent results achieved in color image super-resolution by generative adversarial networks, we propose a depth map super-resolution framework with generative adversarial networks using multiscale attention fusion. Fusion of the color features and depth features at the same scale under the hierarchical fusion attention module effectively measure the guiding effect of the color image on the depth map. The fusion of joint color–depth features at different scales balances the impact of different scale features on the super-resolution of the depth map. The loss function of a generator composed of content loss, adversarial loss, and edge loss helps restore clearer edges of the depth map. Experimental results on different types of benchmark depth map datasets show that the proposed multiscale attention fusion based depth map super-resolution framework has significant subjective and objective improvements over the latest algorithms, verifying the validity and generalization ability of the model.

## 1. Introduction

With the increasing emphasis on security, trustworthy artificial intelligence is on the rise. In trustworthy AI, various 3D applications play a crucial role in scene construction, understanding the relationship between entities and the scene, and reasoning about invisible factors outside the scene. In the research of stereo image technology, the quality of depth maps is very significant because the depth value reflects the spatial position of the object in the scene. However, the resolution of depth maps has been very low due to the limited capture capability of depth sensors. Therefore, depth map super-resolution (SR) has become an urgent problem to be solved.

Due to the limited information contained in a single depth map, the corresponding high-resolution (HR) color image is generally used to guide the super-resolution of depth maps. Conventional methods use filters or Markov Random Fields (MRF) to implement depth map super-resolution with the guidance of the color image. Leveraging the HR color image and the given low-resolution (LR) depth map, Kopf et al. [1] proposed a joint bilateral filter (JBU) which combines a range filter and a spatial filter to produce very good full resolution results. Diebel and Thrun [2] first formulated the depth map SR as a multi-labeling optimization problem based on the MRF model by connecting the color image and the depth map as the balance factor of the smooth term.

In recent years, due to the rapid development of convolutional neural networks, color-guided depth map super-resolution methods based on convolutional neural networks have achieved more remarkable results. Hui et al. [3] proposed a multiscale guided convolutional network (MSG-Net) for depth map super-resolution which complements low-resolution depth features with HR intensity features using a multiscale fusion strategy. Ye et al. [4] constructed a convolutional neural network architecture to learn a binary map of depth edge location from a low-resolution depth map and the corresponding color image, and then proposed a fast edge-guided depth filling strategy to interpolate the missing depth.

However, most color-guided depth map super-resolution methods use color images directly. How to quantitatively measure the guiding effect of color images on depth map super-resolution lacks the attention of researchers. In this paper, we propose a depth map super-resolution framework that uses hierarchical attention fusion modules to measure the guidance of color features on depth features. Inspired from the recent emergence of excellent color image super-resolution generative adversarial networks such as SRGAN [5] and ESRGAN [6], our framework uses relativistic standard generative adversarial networks as the backbone. In particular, a loss model generator that includes content loss, adversarial loss, and edge loss helps the proposed generative adversarial networks produce clearer edges of the depth map.

Our main contributions are as follows: (1) We propose a depth map super-resolution framework with multiscale attention fusion based generative adversarial networks to quantitatively measure the effectiveness of color images as a guide to depth map super-resolution. (2) The hierarchical color–depth attention fusion module measures the guidance of the color image on the depth map super-resolution and generates fused features of various scales. (3) The multiscale fused feature balance module evaluates the correlation between scales and fused features, and integrates fused color–depth features of various scales proportionally. (4) A loss function consisting of content loss, adversarial loss, and edge loss helps our method produce clearer edges of the depth map.

We organize the remainder of this paper as follows. After a brief review of related literature in Section 2, we present the framework and introduce the details of our method in Section 3. In Section 4, we conduct an ablation study and comparison experiments on the benchmark depth map datasets, and discuss the performance of our method compared to other methods. Finally, we conclude the whole paper in Section 6.

## 2. Related Works

In this section, we introduce color-guided depth map super-resolution and color image super-resolution generative adversarial networks methods.

### 2.1. Conventional Color-Guided Depth Map Super-Resolution

Conventional color-guided depth map super-resolution methods can be divided into three categories: filter based methods, MRF based methods, and sparse representation based methods.

Filter-based methods [1,7,8,9,10,11,12,13] aim to construct upsampling filters to enhance the depth map resolution with the guidance of the registered color image. Leveraging the HR color image and the given low-resolution depth map, Kopf et al. [1] proposed a joint bilateral filter (JBU) which combines a range filter and a spatial filter to produce very good full-resolution results. In [8], Kim et al. proposed a modified JBU called JABDU that computes each depth value as the average of neighboring pixels weighted by color and depth intensity filters, which are formulated as an adaptive smoothing parameter and a control parameter, respectively. Inspirited from the geodesic distance, Liu et al. [9] advanced the resolution of a depth map using geodesic paths to the pixels whose depths are known from the low-resolution ones. A weighted mode filter (WMF) is proposed in [10] by seeking a global mode on the histogram which uses the weight considering color similarity between the reference and neighboring pixels on the color image to upsample the depth map. Furthermore, Fu et al. [11] incorporated a noise-aware filter (NAF) into a WMF. In order to reduce the artifacts such as texture copy and edge discontinuities, Lo et al. [12] constructed a joint trilateral filtering (JTF) algorithm for depth image SR considering spatial distance, color difference, and local depth gradient simultaneously to better preserve the contour information. Filter-based depth map SR methods can remove the external and internal noise of the depth map, and simultaneously preserve contour features of it. However, with the color image guiding them, these methods can produce texture copy artifacts in smooth regions of the depth map which correspond to richly textured regions of the color image.

Optimization-based single depth map SR methods can be generally divided into two classes: Markov Random Fields (MRF) [2,14,15,16,17,18,19] based algorithms and Sparse Representation based algorithms. Diebel and Thrun [2] first formulate the depth map SR as a multi-labeling optimization problem based on the MRF model. The method in [15] extends the MRF model by presenting a novel data term allowing for adaptive pixel-wise determination of an appropriate depth reference value. In [14], Zuo et al. proposed a method to quantitatively measure the inconsistency between the depth edge map and the color edge map and explicitly embedded the measurement into the smoothness term of the MRF model. Utilizing the edges of the low-resolution depth image through a Markov Random Fields optimization in a patch synthesis based manner, Xie et al. [17] constructed a high-resolution edge map to guide the upscaling of the depth map. By solving an MRF labeling optimization problem, Lo et al. presented a learning-based depth map super-resolution framework in [12] which exhibits the capability of preserving the edges of range data while suppressing the artifacts of texture copy due to color discontinuities. Compared with filter-based methods, optimization-based methods are more robust to noise. For the condition that the edges in a depth map correspond to the smooth region of the color image, blurred edge artifacts can be generated in the SR process due to the inconsistency between the edges of the depth map and color image at the same location.

Many sparse representation-based depth map SR methods [20,21,22,23,24,25] have been proposed in the last few years. They usually cut HR color images and LR depth maps into patches and bind them in pairs to train a dictionary. The depth map SR solutions can be represented as a linear combination of elements in the learned dictionary. Ferstl et al. [21] presented a variational sparse representation approach by using a dictionary of edge priors which learned from an external database of high- and low-resolution examples. In [22], Xie et al. reconstructed the corresponding HR depth map through a robust coupled dictionary learning method with locality coordinate constraints. Simultaneously, an adaptively regularized shock filter is introduced to reduce sharpening of the contours and the jagged noise. Zhang et al. proposed a dual sparsity model based single depth map SR method by combining the analysis model and synthesis model in [24]. As this category of methods utilizes amounts of depth map patches in the training stage, the performance of them heavily relies on the selection of external datasets. In addition, few representation-based depth map SR methods suffer from blurring edge artifacts on the depth edges or the overlapping regions of adjacent patches similar to the optimization-based depth map SR methods.

Single depth map SR methods can achieve a promising performance in preserving depth contour while alleviating the noise of the depth map. However, they can produce texture copy artifacts and blurring edge artifacts derived from the depth discontinuities that are not consistent with color discontinuities at the corresponding position.

### 2.2. Neural-Networks-Based Depth Map Super-Resolution

Depth map super-resolution methods based on neural networks have achieved promising success [3,4,26,27]. The authors of [3] proposed a multiscale guided convolutional network (MSG-Net) for depth map super-resolution which complements low-resolution depth features with HR intensity features using a multiscale fusion strategy. Ye et al. [4] constructed a convolutional neural network architecture to learn a binary map of depth edge location from a low-resolution depth map and corresponding color image. They then proposed a fast edge-guided depth filling strategy to interpolate the missing depth constrained by the acquired edges to prevent predicting across the depth boundaries. Wang et al. [26] proposed a novel depth upsampling framework based on deep edge-aware learning which firstly learns edge information of depth boundaries from the known LR depth map and its corresponding high-resolution (HR) color image as reconstruction cues. Then, two depth restoration modules, i.e., a fast depth filling strategy and a cascaded restoration network, are proposed to recover an HR depth map by leveraging the predicted edge map and the HR color image. In [28], Zuo et al. proposed a novel DCNN to progressively reconstruct the high-resolution depth map guided by the intensity image. Specifically, the multiscale intensity features are extracted to provide guidance for the refinement of depth features as their resolutions are gradually enhanced. In [27] by Zuo et al., a novel depth-guided affine transformation is used to filter out the unrelated intensity features, which is further used to refine the depth features. Since the quality of initial depth features is low, the depth-guided intensity features filtering and the intensity-guided depth features refinement are iteratively performed, which progressively promotes the effects of such tasks.

Images at different scales contain different feature information [3]. However, the guidance of color image features at different scales on depth map super-resolution is not equal. It is not appropriate to cascade or link them directly. As far as we know, quantitative evaluation of the correlation between the scales of features and depth map super-resolution is a topic that has not been discussed. In this article, we use a multiscale fused feature balance module to measure the correlations between different scale features and depth map super-resolution, and further fuse the color–depth features at different scales proportionally.

### 2.3. Generative Adversarial Network Based Color Image Super-Resolution

Super-resolution methods for color images based on generative adversarial networks [5,6,29,30,31] generate realistic high-resolution color images by means of successive iterations of mutual adversaries between generators and discriminators.

Denton et al. [29] introduced a generative parametric model capable of producing high-quality samples of natural images. It uses a cascade of convolutional networks within a Laplacian pyramid framework to generate images in a coarse-to-fine fashion. At each level of the pyramid, a separate generative convnet model is trained using the generative adversarial networks (GAN) approach (Goodfellow et al.). Samples drawn from their model are of significantly higher quality than alternate approaches. The key idea of [30] is to grow both the generator and discriminator progressively: starting from a low resolution and adding new layers that model increasingly fine details as training progresses. This both speeds the training up and greatly stabilizes it to produce images of unprecedented quality. Ledig et al. [5] presented SRGAN, a generative adversarial network (GAN) for image super-resolution (SR). To our knowledge, it is the first framework capable of inferring photo-realistic natural images for 4× upscaling factors. To achieve this, they propose a perceptual loss function which consists of an adversarial loss and a content loss. The adversarial loss pushes their solution to the natural image manifold using a discriminator network that is trained to differentiate between the super-resolved images and original photo-realistic images. The super-resolution generative adversarial network (SRGAN) is a seminal work that is capable of generating realistic textures during single image super-resolution. However, the hallucinated details are often accompanied with unpleasant artifacts. To further enhance the visual quality, Wang et al. [6] thoroughly studied three key components of SRGAN, network architecture, adversarial loss, and perceptual loss, and improve each of them to derive an enhanced SRGAN (ESRGAN).

Some excellent methods for color image super-resolution generative adversarial networks have emerged. However, they produce more artifacts and textures. Obviously, these networks are not suitable for depth map super-resolution. Therefore, considering the sharp edges and smooth interior of the depth map, we propose a multiscale attention fusion based super-resolution generative adversarial network for depth maps. In particular, building a generator loss function that includes content loss, adversarial loss, and edge loss facilitates the generation of sharper edges.

## 3. Multiscale Attention Fusion for Depth Map Super-Resolution Generative Adversarial Networks

In this section, we propose a multiscale attention fusion for depth map super-resolution generative adversarial networks.

### 3.1. Framework

**Figure 1:**
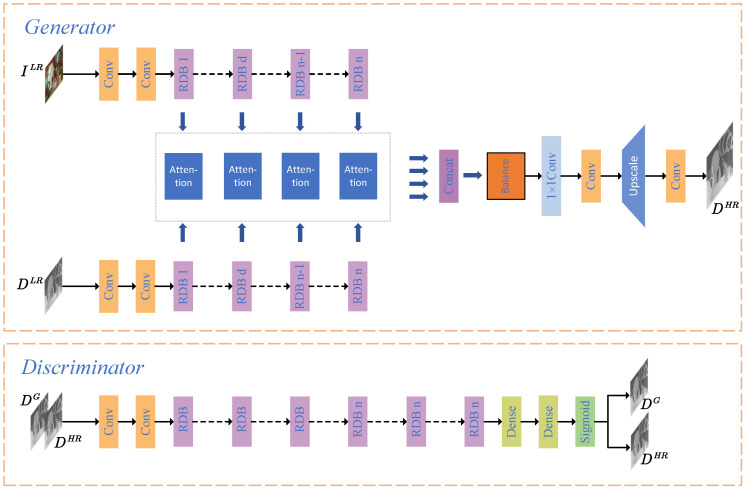
Framework of the multiscale attention fusion for depth map super-resolution generative adversarial networks. $D_{LR}$ and $I_{LR}$ are the low-resolution depth map and the corresponding downsampled color image. $D_{HR}$ is the high-resolution depth map generated by the generator of our proposed GAN. Furthermore, $D_{G}$ is the ground truth depth map.

The framework of our proposed method is demonstrated in Figure 1. In Figure 1, our goal is to generate a precise high-resolution depth estimation $D_{HR}$ of the ground truth $D_{G}$. The generator consists of four parts: a multiscale color and depth feature extraction module, a hierarchical feature attention fusion module, a multiscale fused feature balance module, and a super-resolution module. The multiscale color and depth feature extraction module extracts different scale features using a low-resolution depth map and the corresponding color image as inputs. It consists of two convolutional layers and *n* residual dense blocks (RDBs), where *n* is the scale of feature extraction. The settings of RDBs are consistent with those in [32]. The depth feature and color feature passing through the *i*th RDB are represented as $F_{D}^{i}$ and $F_{I}^{i}$, respectively. Previous methods have used these to directly concatenate depth features and color features together. However, the guidance of color features on depth features should not be just a simple link. How to quantitatively measure the guidance of color features on depth features has become a key issue. In this article, we propose using an attention module to measure the guiding effect of color features on depth features. $F_{D}^{i}$ and $F_{I}^{i}$ form a color–depth fused feature $F_{f}^{i}$ at the *i*th scale through the attention module. In this way, we obtain color–depth fused features $F_{f}^{1}$, $F_{f}^{2}$, *…*, $F_{f}^{n}$ at *n* scales. Images at different scales contain different geometric structures. The contribution of fused features at different scales to depth map super-resolution is not equal. We input $F_{f}^{1}$-$F_{f}^{n}$ into the multiscale fused feature balance module to evaluate the correlations between the scales and fused features, and obtain a final fused feature $F_{f}$. We choose UPNet as [32] as the super-resolution module of the generator. The high-resolution depth map $D_{HR}$ is generated by $F_{f}$ through UPNet.

### 3.2. Hierarchical Color and Depth Attention Fusion Module

**Figure 2:**
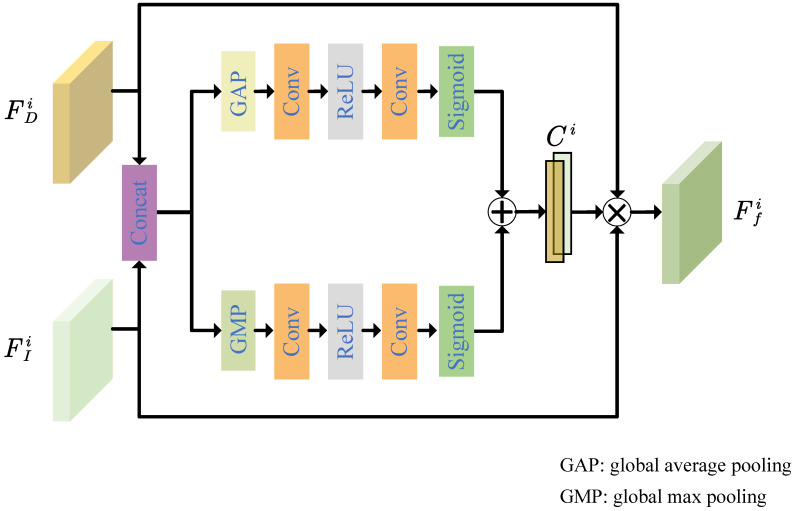
Hierarchical color and depth attention fusion module.

The details of the proposed hierarchical color and depth attention fusion module are shown in Figure 2. Before inputting them into the module, we first concatenate the color feature $F_{I}^{i}$ and the depth feature $F_{D}^{i}$ at the *i*th scale to form the merged feature $F_{C}^{i}$. Then, $F_{C}^{i}$ is fed into global average pooling and global sum pooling, respectively. Global average pooling and global sum pooling are followed by two convolutional layers and one ReLU, respectively. By processing the convolutional features through the sigmoid function, two coefficient matrices are obtained. By adding and splitting the coefficient matrices in place we can obtain the weight coefficient vector $C_{i}$ of $F_{I}^{i}$ and $F_{D}^{i}$ as in Equation (1).

(1) $$\begin{array}{c} \begin{array}{c} C^{i}=f_{att}\left(F_{D}^{i},F_{I}^{i}\right), \end{array} \end{array}$$

where $f_{att}$ denotes the color and depth attention fusion module. Multiplying $F_{I}^{i}$ and $F_{D}^{i}$ element-wise by the corresponding coefficient vector $C_{i}$, we obtain the fused color–depth fused feature $F_{f}^{i}$ at the *i*th scale as in Equation (2).

(2) $$\begin{array}{c} \begin{array}{c} F_{f}^{i}=\left[F_{D}^{i},F_{I}^{i}\right]C^{i} \end{array} \end{array}$$

### 3.3. Multiscale Fused Feature Balance Module

After obtaining the color–depth fused features $\left\{F_{f}^{i}\right\}$ using the *n* attention modules, we concatenate these features and denote them as $F_{f}^{C}$.

(3) $$\begin{array}{c} \begin{array}{c} F_{f}^{C}=\left[F_{f}^{1},F_{f}^{2},\ldots ,F_{f}^{i},\ldots ,F_{f}^{n}\right] \end{array} \end{array}$$

**Figure 3:**
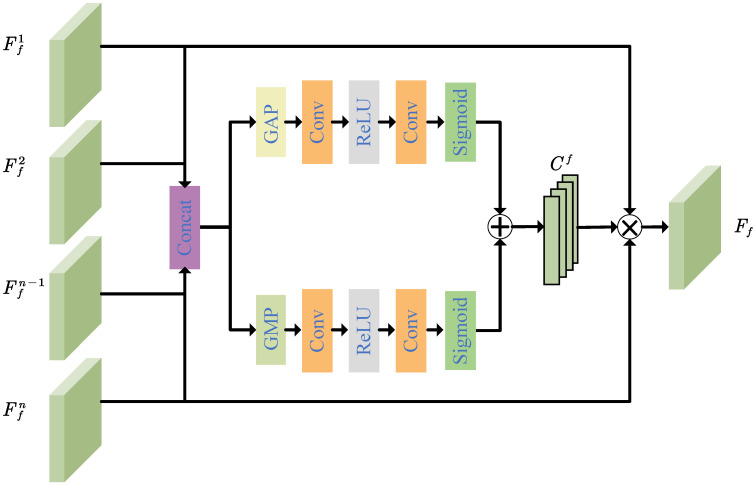
Details of multiscale fused feature balance module.

Then $F_{f}^{C}$ is fed to the multiscale fused feature balance module.

(4) $$\begin{array}{c} \begin{array}{c} W_{f}=f_{bal}\left(F_{f}^{C}\right) \end{array} \end{array}$$

where $W_{f}$ is a vector of balance factors and $f_{bal}$ is the multiscale fused feature balance module. The multiscale fused feature balance module is used to evaluate the correlations between the scales and the fused features as shown in Figure 3. It consists of two branches. These two branches start with a global average pooling and a global sum pooling, separately, followed by two convolutional layers, a ReLU layer, and a sigmoid function. $F_{f}^{C}$ generates two weight coefficient matrices through these two branches. The two weight coefficient matrices are summed and separated to obtain $W_{f}$. The balanced multiscale color–depth feature $F_{f}$ is generated by multiplying the concatenated sequence $F_{f}^{C}$ with the corresponding balance factor vector as in Equation (5).

(5) $$\begin{array}{c} \begin{array}{c} F_{f}=F_{f}^{C}W_{f} \end{array} \end{array}$$

### 3.4. Relativistic Standard Generative Adversarial Networks

In the standard GAN, the discriminator outputs the probability that the input image is real to determine whether the input is real or fake. The type of GAN can be defined with the discriminator. In general, the discriminator loss of a standard GAN with the assumption of cross-entropy loss can be expressed as follows:

(6) $$\begin{array}{c} \begin{array}{c} L_{D}=E_{x_{r}∼P}\left[f_{1}\left(D\left(x_{r}\right)\right)\right]+E_{x_{f}∼Q}\left[f_{2}\left(D\left(x_{f}\right)\right)\right] \end{array} \end{array}$$

where $x_{r}$ and $x_{f}$ indicate the real depth map and the fake one, respectively. The adversarial loss of the generator is expressed as

(7) $$\begin{array}{c} \begin{array}{c} L_{G}=E_{x_{r}∼P}\left[g_{1}\left(C\left(x_{r}\right)\right)\right]+E_{x_{f}∼Q}\left[g_{2}\left(C\left(x_{f}\right)\right)\right], \end{array} \end{array}$$

where

(8) $$\begin{array}{c} \begin{array}{cc} & f_{1}\left(D\left(x\right)\right)=g_{2}\left(D\left(x\right)\right)=-\log\left(D\left(x\right)\right)\\ & f_{2}\left(D\left(x\right)\right)=g_{1}\left(D\left(x\right)\right)=-\log\left(1-D\left(x\right)\right) \end{array} \end{array}$$

$D\left(x\right)$ is the activation function of the non-tranformed layer $C\left(x\right)$ as in Equation (9).

(9) $$\begin{array}{c} \begin{array}{c} D\left(x\right)=\mathrm{sigmoid}\left(C\left(x\right)\right) \end{array} \end{array}$$

In the discriminator, $D_{G}$ and $D_{HR}$ are input as $x_{r}$ and $x_{f}$. Because the gradient of $g_{1}$ is 0, only half of the generator is involved during the training process.

In this paper, we adopt the relativistic standard GAN (RGAN) [33] structure to achieve the full participation of the generator. The discriminator of RGAN estimates the probability that the given real data is more realistic than a randomly sampled fake data. It is denoted as Equation (10).

(10) $$\begin{array}{c} \begin{array}{c} D\left(x_{r},x_{f}\right)=\mathrm{sigmoid}\left(C\left(x_{r}\right)-C\left(x_{f}\right)\right) \end{array} \end{array}$$

Correspondingly, the loss of the discriminator is expressed as follows:

(11) $$\begin{array}{c} \begin{array}{c} L_{D}=-E_{\left(x_{r},x_{f}\right)∼\left(P,Q\right)}\left[\log\left(\mathrm{sigmoid}\left(C\left(x_{r}\right)-C\left(x_{f}\right)\right)\right)\right]-E_{\left(x_{r},x_{f}\right)∼\left(P,Q\right)}\left[\log\left(1-\mathrm{sigmoid}\left(C\left(x_{f}\right)-C\left(x_{r}\right)\right)\right)\right] \end{array} \end{array}$$

The adversarial loss of the generator is expressed as Equation (14).

(12) $$\begin{array}{c} \begin{array}{c} L_{G}=-E_{\left(x_{r},x_{f}\right)∼\left(P,Q\right)}\left[\log\left(1-\mathrm{sigmoid}\left(C\left(x_{r}\right)-C\left(x_{f}\right)\right)\right)\right]-E_{\left(x_{r},x_{f}\right)∼\left(P,Q\right)}\left[\log\left(\mathrm{sigmoid}\left(C\left(x_{f}\right)-C\left(x_{r}\right)\right)\right)\right] \end{array} \end{array}$$

The discriminator extracts features using the RDBs, and then performs a discriminant classification using the sigmoid function to determine whether the input depth map is fake or real. Compared to the standard GAN, the relativistic GAN can generate high-resolution depth maps from relatively small samples. Furthermore, the training time to achieve optimal performance is significantly reduced.

Due to the fact that depth maps are a kind of piece-wise smooth images, they are characterized by sharp edges and smooth interiors. Conventional GAN-based color image super-resolution methods that only use mean squared error (MSE) as content loss are not suitable for depth map super-resolution. In order to improve the edge sharpness of the generated high-resolution depth maps, we propose a loss function consisting of content loss, adversarial loss, and edge loss, which is expressed by Equation (13).

(13) $$\begin{array}{c} \begin{array}{c} {\tilde{L}}_{G}=L_{C}+\mu L_{G}+\gamma L_{e} \end{array} \end{array}$$

where

(14) $$\begin{array}{c} \begin{array}{c} L_{C}=\frac{1}{N}\sum_{i=1}^{N}{\left(D^{HR}-D^{G}\right)}^{2}\\ L_{e}=\frac{1}{N}\sum_{i=1}^{N}{\left(e^{HR}-e^{G}\right)}^{2} \end{array} \end{array}$$

μ and γ are the scale factors which balance the adversarial loss and the edge loss.

## 4. Experimental Results

### 4.1. Parameter Setting

We train our network with 80 color and depth pairs. In the training dataset, 52 color–depth pairs are from the Middlebury dataset and others are from the MPI Sintel depth dataset. The color images are downsampled to the images of corresponding scale factor by interval interpolation. The patch size is 128 × 128 and the batch size is 16. To enrich data diversity, we flip the patches horizontally and vertically, and rotate them by 90${}^{\circ }$. The kernel size of all convolution layers is 3 and the channels of the feature map number 64. We take ReLU function as the activation function after all convolution layers. Adam is set as the optimizer with $\beta_{1}=0.9$ and $\beta_{2}=0.999$. Our proposed method is implemented on two Nvidia RTX 2080ti GPUs. We train our network for 1000 epochs, and the initial learning rate is $10^{-4}$ and halved every 200 epochs.

### 4.2. Datasets

**Training Datasets**

In the training phase, we use two datasets, the Middlebury datasets and the MPI Sintel depth dataset. The Middlebury datasets [34] are a stereo dataset widely used in applications related to stereo matching, 3D reconstruction, and stereo quality evaluation. It consists of five versions constructed in different years, namely the 2001 dataset, 2003 dataset, 2005 dataset, 2006 dataset, 2014 dataset, and 2021 moving dataset. We randomly selected 52 datasets from the Middlebury datasets of different view, size, illuminations, and exposures.

The MPI Sintel depth dataset [35] is a synthetic stereo dataset which provides naturalistic video sequences. The depth values in the MPI Sintel depth dataset are returned from Blender with an additional Z-buffer pass, similar to the optical flow.

**Testing Datasets**

Among the Middlebury Stereo Datasets [34], we use six color–depth pairs as the testing samples. They are Art, Books, Moebius, Reindeer, Laundry, and Dolls. To better demonstrate the effectiveness of our method, we also conduct experiments on the Multi-view depth (MVD) test sequences [36] and ToFMark dataset [37].

The Multi-view depth (MVD) test sequences consist of multi-view video sequences and corresponding pixel-by-pixel depth information to support flexible synthesis of virtual views during rendering. It is widely used in studies on 3D applications such as free-view video, binocular stereoscopic video, and naked eye 3D stereo video, making it the most promising form of 3D video data representation today.

ToFMark dataset containes three real-scene depth maps captured by ToF sensors. The low-resolution depth maps in it were acquired using the PMD Nano ToF camera with a resolution of 120 × 160, and the high-resolution color images were acquired using the CMOS camera with a resolution of 810 × 610.

### 4.3. Evaluation Metrics

For reconstructed and enhanced images, many studies have proposed many objective evaluation criteria [38,39]. In this paper, we take three metrics to evaluate the performance of our proposed method in depth map super-resolution. They are RMSE, MAD, and PSNR.

RMSE stands for root mean squared error, as in Equation (15).

(15) $$\begin{array}{c} \begin{array}{cc} \mathrm{RMSE} & =\sqrt{\frac{1}{N}\sum_{i=1}^{N}{\left(D^{HR}-D^{G}\right)}^{2}} \end{array} \end{array}$$

MAD represents Mean Absolute Difference, described by Equation (16).

(16) $$\begin{array}{c} \begin{array}{cc} \mathrm{MAD} & =\frac{1}{N}\sum_{i=1}^{N}\left|D^{HR}-D^{G}\right| \end{array} \end{array}$$

Peak Signal to Noise Ratio (PSNR) is also a commonly used objective criterion for evaluating image quality,

(17) $$\begin{array}{c} \begin{array}{c} \mathrm{PSNR}=10\times \log_{10}\left(\frac{{\left(2^{n}-1\right)}^{2}}{\mathrm{MSE}}\right) \end{array} \end{array}$$

where MSE is mean squared error in Equation (18), which is the square of RMSE.

(18) $$\begin{array}{c} \begin{array}{cc} \mathrm{MSE} & =\frac{1}{N}\sum_{i=1}^{N}{\left(D^{HR}-D^{G}\right)}^{2} \end{array} \end{array}$$

### 4.4. Comparison of Different Numbers of RDBs

In this subsection, we explore the correlation between the quantity of scales in the multiscale fusion attention module on the performance of the depth map super-resolution. We tested the experimental results on the Middlebury datasets for four quantities of RDBs: 10, 16, 20, and 22. The selection type of GAN is RGAN and the loss of the generator is set to content loss + edge loss. The experimental results are shown in Table 1. We can see that as the quantity of RDBs increases, the RMSE of the generated depth map decreases. However, after the number of scales exceeds 16, the effect of depth map super-resolution is not significantly improved. Considering the increased storage and computing consumption, we believe that 16 is the most reasonable number of RDBs.

### 4.5. Comparison of GAN Types

In this subsection, we compare the depth map SR results with different kinds of GANs. Table 2 demonstrates the experimental results of our proposed method with GAN and RGAN. We choose 16 as the number of scales in the multiscale fusion attention module and MSE + edge loss as the loss of generator. It can be seen that the RMSE of our method with RGAN is better than that with GAN. This shows that our method uses RGAN to generate high-resolution depth maps that are closer to the real depth maps.

### 4.6. Comparison of Generator Losses

**Figure 4:**
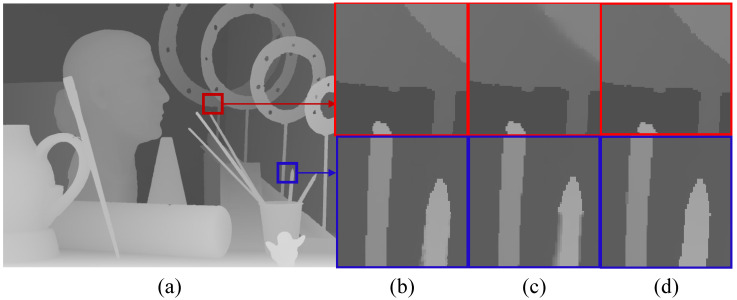
Visual comparison of Art with cropped zoomed regions (scaling factor = 4). They are (**a**) depth map, (**b**) ground truth, (**c**) our method with MSE loss, (**d**) our method with MSE + edge loss.

In this subsection, we compare the experimental results with generator losses of MSE loss and MSE loss + edge loss, besides adversarial loss, to verify the necessity of edge loss. The general color image super-resolution generative adversarial networks are reconstructed based on MSE loss, which can obtain closer objective experimental results to the ground truth. However, the visual performance of the image generated in this way is not perceptually the closest to ground truth. Therefore, we propose a generator loss function that includes an edge loss for the characteristics of the clear edges and the smooth interior of the depth maps. As shown in Table 3, the RMSE of the depth maps generated by RGAN with edge loss is very close to the RMSE of those generated by a network containing only MSE. However, Figure 4 shows the comparison of two sets of super-resolution results on Art. We can see that the network containing edge loss generates high-resolution depth maps with clearer edges compared to the GAN containing only MSE, thereby verifying the effectiveness of edge loss in generating perceptually high-quality depth maps.

### 4.7. Experimental Results on Middlebury Datasets

Our baseline state-of-the-art methods are joint bilateral upsampling (JBU) [1], noise-aware filter (NAF) [40], anisotropic diffusion [41], Markov random field (MRF) [2], guided image filtering (GIF) [42], SRF from [43], edge weighted NLM regularization (Edge) [44], joint geodesic filtering (JGF) [9], total generalized variation (TGV) from [37], four deep learning method SRCNNs from [45], deep joint image filter (DJIF) from [46], deep edge-aware network (DSR) from [26] and cross-guided network for depth map enhancement (CGN) from [27], two GAN-based color image super-resolution methods for a super-resolution generative adversarial network (SRGAN) from [5], enhanced SRGAN (ESRGAN) from [6], and dictionary learning method JESR from [20] that are used in comparison to evaluate the performance of our method. We set the number of RDBs to 16 and the type of GAN to RGAN in our method. The depth map upscaling factors are set to 2, 4, 8, and 16.

In Table 4 and Table 5, we can see that both DSR and CGN obtain top-ranked experimental results. Compared with the other two color image super-resolution GAN methods, our proposed method gains the lowest RMSE and MAD. This is because SRGAN and ESRGAN are designed for color images with a structure that produces more texture. However, they are not suitable for the internal smoothing properties of depth maps.

**Figure 5:**
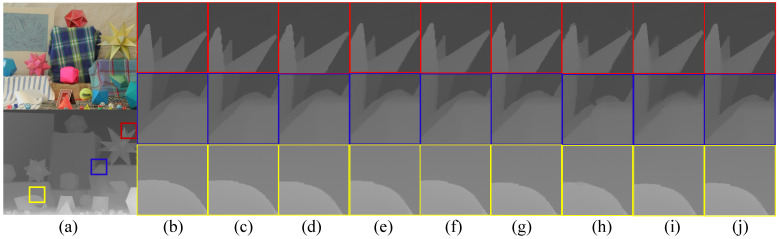
Visual comparison of Moebius with cropped zoomed regions (scaling factor = 4). They are (**a**) depth map, (**b**) ground truth, (**c**) JBU [1], (**d**) MRF [2], (**e**) TGV [37], (**f**) ESRGAN [6], (**g**) DJIF [46], (**h**) DSR [26], (**i**) CGN [27], and (**j**) ours.

Figure 5 shows the visual comparison of the state-of-the-art baselines with our method. It can be seen that our method produces clearer and sharper edges, and avoids artifacts of blurred edges and texture transfer.

### 4.8. Experimental Results on Real Datasets

Since depth maps are acquired by depth sensors in real scenes, we not only compare experimental results on the Middlebury datasets, but also conduct experiments and comparisons on real scene depth map datasets. In this article, we selected the ToFMark dataset captured by the ToF sensor and the multi-view depth (MVD) test sequences [36] as the test sets. Our comparison methods are bicubic, joint geodesic filtering (JGF) [9], total generalized variation (TGV) from [37], SRGAN from [5], enhanced SRGAN (ESRGAN) from [6], deep joint image filter (DJIF) from [46], deep edge-aware network (DSR) from [26], and cross-guided network for depth map enhancement (CGN) from [27]. The depth map upscaling factors are set to 2, 4, 8, and 16.

Table 6 and Table 7 demonstrate the quantitative depth upsampling results on ToFMark dataset and MVD dataset, respectively. Our proposed method shows the best objective performance over other the state-of-the-art methods.

## 5. Discussion

In this section, we briefly discuss our proposed method and the directions we can focus on in the future. In the edge region of the depth map, the introduced color image corresponds to a smooth region, resulting in the generated high-resolution depth map with occasional edge blurring. In the future, we will focus on introducing color image edges aligned with the edges of the depth map into the framework to achieve more accurate depth map super-resolution as well as to generate sharper edges.

## 6. Conclusions

In this paper, we propose a multiscale attention fusion based depth super-resolution generative adversarial networks for 3D reconstruction in trustworthy AI. Specifically, a hierarchical color–depth attention fusion module measures the guidance of the color image on the depth map super-resolution and generates fused features of various scales. The multiscale fused feature balance module evaluates the correlation between scales and fused features, and integrates fused color–depth features at different scales in a proportional manner. By constructing a loss function model consisting of content loss, adversarial loss, and edge loss, our proposed generative adversarial networks produce high-resolution depth maps with sharper edges. The robustness and generalization of the model is demonstrated by extensive experiments that show satisfactory subjective and objective results of our proposed method on several types of depth map datasets.

## Figures and Tables

**Table 1:** Quantitative comparison of depth upsampling results (RMSE) on the Middlebury datasets regarding the quantity of RDBs.

Algorithm	Art				Book				Moebius				Reindeer				Laundry				Dolls			
	2×	4×	8×	16×	2×	4×	8×	16×	2×	4×	8×	16×	2×	4×	8×	16×	2×	4×	8×	16×	2×	4×	8×	16×
RDB_10	1.19	2.40	3.13	3.84	0.79	1.26	1.58	2.31	0.52	0.81	1.27	1.69	1.45	1.71	2.48	3.35	1.32	1.75	2.16	3.09	0.87	1.13	1.37	1.69
RDB_16	0.81	2.15	2.81	3.47	0.49	0.94	1.30	1.83	0.29	0.58	0.95	1.52	1.28	1.49	2.18	3.03	1.11	1.47	1.96	2.80	0.61	0.84	1.03	1.46
RDB_20	0.79	2.13	2.78	3.43	0.46	0.91	1.27	1.80	0.26	0.55	0.90	1.48	1.24	1.45	2.16	2.98	1.06	1.45	1.92	2.78	0.59	0.82	0.98	1.43
RDB_22	**0.78 **	**2.10**	**2.76**	**3.39**	**0.44**	**0.89**	**1.25**	**1.79**	**0.23**	**0.52**	**0.87**	**1.46**	**1.21**	**1.42**	**2.15**	**2.97**	**1.03**	**1.44**	**1.90**	**2.75**	**0.56**	**0.81**	**0.96**	**1.38**

**Table 2:** Quantitative comparison of depth upsampling results (RMSE) on the Middlebury datasets regarding GAN types.

Algorithm	Art				Book				Moebius				Reindeer				Laundry				Dolls			
	2×	4×	8×	16×	2×	4×	8×	16×	2×	4×	8×	16×	2×	4×	8×	16×	2×	4×	8×	16×	2×	4×	8×	16×
GAN	0.96	2.37	3.02	3.68	0.61	1.25	1.58	2.19	0.46	0.73	1.21	1.74	1.50	1.81	2.49	3.27	1.45	1.75	2.32	3.33	0.92	1.14	1.49	1.87
RGAN	**0.81 **	**2.15**	**2.81**	**3.47**	**0.49**	**0.94**	**1.30**	**1.83**	**0.29**	**0.58**	**0.95**	**1.52**	**1.28**	**1.49**	**2.18**	**3.03**	**1.11**	**1.47**	**1.96**	**2.80**	**0.61**	**0.84**	**1.03**	**1.46**

**Table 3:** Quantitative comparison of depth upsampling results (RMSE) on the Middlebury datasets regarding generator loss.

Algorithm	Art				Book				Moebius				Reindeer				Laundry				Dolls			
	2×	4×	8×	16×	2×	4×	8×	16×	2×	4×	8×	16×	2×	4×	8×	16×	2×	4×	8×	16×	2×	4×	8×	16×
MSE	**0.79**	**2.13**	**2.75**	**3.42**	**0.48**	**0.91**	**1.26**	**1.80**	**0.25**	**0.54**	**0.93**	**1.48**	**1.21**	**1.46**	**2.13**	**3.00**	**1.06**	**1.45**	**1.92**	**2.77**	**0.58**	**0.81**	**1.01**	**1.44**
MSE + Edge	0.81	2.15	2.81	3.47	0.49	0.94	1.30	1.83	0.29	0.58	0.95	1.52	1.28	1.49	2.18	3.03	1.11	1.47	1.96	2.80	0.61	0.84	1.03	1.46

**Table 4:** Quantitative depth upsampling results (RMSE) on Middlebury datasets.

Algorithm	Art				Book				Moebius				Reindeer				Laundry				Dolls			
	2×	4×	8×	16×	2×	4×	8×	16×	2×	4×	8×	16×	2×	4×	8×	16×	2×	4×	8×	16×	2×	4×	8×	16×
JBU [1]	3.49	5.08	6.26	9.74	1.78	2.50	2.97	5.44	1.50	2.14	2.99	4.29	2.46	3.29	4.08	5.86	2.42	3.08	4.12	5.84	1.38	1.77	2.43	3.30
NAF [40]	3.52	5.10	6.39	10.45	1.85	2.44	3.03	5.76	1.51	2.27	3.01	4.38	2.48	3.36	4.49	6.34	2.49	3.13	4.44	6.20	1.45	1.96	2.83	3.51
AD [41]	4.16	4.88	6.65	9.71	1.66	2.23	2.95	5.28	1.44	2.15	3.11	4.40	2.59	3.35	4.51	6.37	2.51	3.17	4.25	5.96	1.31	1.80	2.69	3.59
MRF [2]	3.74	4.75	6.48	9.92	1.73	2.35	3.17	5.34	1.40	2.11	3.17	4.48	2.58	3.29	4.41	6.26	2.54	3.17	4.19	5.92	1.32	1.79	2.66	3.55
GIF [42]	3.15	4.11	5.73	8.53	1.41	2.03	2.58	3.67	1.15	1.65	2.58	4.12	2.19	2.98	4.44	6.58	1.88	2.60	4.02	5.89	1.18	1.67	2.10	3.24
SRF [43]	2.65	3.89	5.51	8.24	1.06	1.62	2.38	3.41	0.90	1.37	2.06	2.99	1.95	2.84	4.10	5.97	1.61	2.40	3.50	5.24	1.14	1.39	1.98	2.79
Edge [44]	2.58	3.24	4.30	6.03	1.21	1.52	1.93	2.60	0.86	1.27	1.99	2.68	1.96	2.89	3.58	3.99	1.62	2.39	3.22	4.29	1.12	1.32	1.51	2.20
JESR [20]	2.63	3.66	5.13	7.05	1.05	1.59	1.83	2.91	0.87	1.21	1.59	2.24	1.95	2.69	3.55	4.88	1.61	2.34	2.84	4.44	1.13	1.32	1.67	2.25
JGF [9]	3.08	3.94	5.25	7.13	1.32	1.82	2.38	3.49	1.14	1.59	2.34	3.47	2.17	2.78	3.50	4.46	1.87	2.59	3.68	5.24	1.13	1.50	1.98	2.71
TGV [37]	2.60	3.34	4.10	6.43	1.20	1.47	1.82	2.63	0.82	1.22	1.64	2.41	1.80	2.71	3.15	4.60	1.61	2.39	2.64	4.17	1.01	1.31	1.61	2.22
SRCNN [45]	2.63	3.53	5.34	7.68	1.20	1.47	1.84	2.84	0.86	1.20	1.87	2.67	2.07	2.78	3.54	4.86	1.67	2.18	2.78	4.49	1.15	1.33	1.66	2.64
SRGAN [5]	2.02	3.57	4.25	5.90	1.08	1.43	1.85	2.79	0.78	1.23	1.60	2.38	1.86	2.68	3.43	4.37	1.54	2.06	2.75	3.96	1.16	1.37	1.64	2.21
ESRGAN [6]	1.76	3.29	3.86	5.49	0.75	1.37	1.69	2.58	0.65	1.01	1.42	2.12	1.73	2.51	3.19	4.08	1.45	1.99	2.52	3.72	0.97	1.25	1.48	2.02
DJIF [46]	1.83	3.46	4.07	4.70	0.77	1.50	1.78	2.61	0.56	1.04	1.47	2.09	2.15	2.59	3.24	4.12	2.04	2.23	2.86	3.88	0.91	1.16	1.45	1.94
DSR [26]	1.41	3.03	3.59	4.02	0.63	1.36	1.62	2.38	0.48	0.85	1.29	1.94	1.52	1.98	2.82	3.89	1.64	1.97	2.41	3.56	0.86	1.04	1.27	1.77
CGN [27]	1.27	2.91	3.46	3.88	0.68	1.25	1.55	2.16	0.33	0.79	1.13	1.71	1.49	1.70	2.65	3.62	1.48	1.72	2.35	3.19	0.75	1.02	1.25	1.73
Ours	**0.81**	**2.15**	**2.81**	**3.47**	**0.49**	**0.94**	**1.30**	**1.83**	**0.29**	**0.58**	**0.95**	**1.52**	**1.28**	**1.49**	**2.18**	**3.03**	**1.11**	**1.47**	**1.96**	**2.80**	**0.61**	**0.84**	**1.03**	**1.46**

**Table 5:** Quantitative depth upsampling results (MAD) on Middlebury datasets.

Algorithm	Art				Book				Moebius				Reindeer				Laundry				Dolls			
	2×	4×	8×	16×	2×	4×	8×	16×	2×	4×	8×	16×	2×	4×	8×	16×	2×	4×	8×	16×	2×	4×	8×	16×
JBU [1]	0.72	1.13	1.95	3.47	0.30	0.41	0.69	1.21	0.31	0.41	0.69	1.24	0.53	0.65	0.94	2.06	0.43	0.64	1.12	2.03	0.33	0.44	0.63	1.18
NAF [40]	0.72	1.24	1.98	3.68	0.30	0.40	0.67	1.24	0.31	0.41	0.61	1.26	0.54	0.65	0.98	2.04	0.45	0.69	1.13	2.01	0.31	0.45	0.66	1.27
AD [41]	0.75	1.22	2.06	4.29	0.33	0.45	0.85	1.54	0.35	0.45	0.74	1.56	0.50	0.64	1.09	2.17	0.48	0.70	1.05	2.75	0.38	0.47	0.81	1.40
MRF [2]	0.80	1.29	2.15	4.25	0.36	0.47	0.86	1.58	0.37	0.43	0.70	1.50	0.52	0.62	1.04	1.96	0.51	0.79	1.10	2.29	0.30	0.46	0.88	1.35
GIF [42]	0.63	1.01	1.70	3.46	0.22	0.35	0.58	1.14	0.23	0.37	0.59	1.16	0.42	0.53	0.88	1.80	0.38	0.52	0.95	1.90	0.28	0.35	0.56	1.13
SRF [43]	0.46	0.97	1.83	3.44	0.15	0.32	0.59	1.12	0.14	0.32	0.51	1.10	0.30	0.55	1.04	1.85	0.23	0.54	1.06	1.99	0.20	0.35	0.56	1.13
Edge [44]	0.41	0.65	1.03	2.11	0.17	0.30	0.56	1.03	0.18	0.29	0.51	1.10	0.20	0.37	0.63	1.28	0.17	0.32	0.54	1.14	0.16	0.31	0.56	1.05
JESR [20]	0.45	0.76	1.51	2.98	0.15	0.27	0.48	0.90	0.16	0.30	0.44	1.01	0.31	0.47	0.69	1.42	0.23	0.50	0.96	1.47	0.20	0.32	0.51	0.92
JGF [9]	0.29	0.47	0.78	1.54	0.15	0.24	0.43	0.81	0.15	0.25	0.46	0.80	0.23	0.38	0.64	1.09	0.21	0.36	0.64	1.20	0.19	0.33	0.59	1.06
TGV [37]	0.45	0.65	1.17	2.30	0.18	0.27	0.42	0.82	0.18	0.29	0.49	0.90	0.32	0.49	1.03	3.05	0.31	0.55	1.22	3.37	0.21	0.33	0.70	2.20
SRCNN [45]	0.22	0.53	0.77	2.13	**0.09**	0.22	0.40	0.79	**0.10**	0.22	0.42	0.89	0.32	0.47	0.68	1.77	0.24	0.50	0.96	1.54	0.23	0.33	0.57	1.09
SRGAN [5]	0.19	0.48	0.70	2.05	0.16	0.28	0.40	0.74	0.15	0.26	0.47	0.81	0.27	0.46	0.65	1.19	0.28	0.44	0.60	1.15	0.20	0.31	0.53	0.88
ESRGAN [6]	0.15	0.36	0.62	1.69	0.14	0.21	0.37	0.65	0.14	0.23	0.42	0.76	0.24	0.43	0.60	1.08	0.23	0.39	0.52	1.08	0.15	0.27	0.48	0.75
DJIF [46]	0.16	0.38	0.68	1.83	0.18	0.25	0.39	0.68	0.19	0.22	0.40	0.73	0.23	0.39	0.52	1.04	0.20	0.30	0.53	1.12	0.18	0.28	0.45	0.79
DSR [26]	0.13	0.31	0.57	1.46	0.15	0.22	0.34	0.61	0.12	0.20	0.37	0.66	0.21	0.37	0.50	0.96	0.15	0.28	0.46	1.07	0.15	0.24	0.42	0.69
CGN [27]	0.11	0.25	0.48	1.39	0.12	0.18	0.30	0.57	0.13	0.18	0.35	0.62	0.18	0.35	0.46	0.83	0.12	0.26	0.43	1.05	0.14	0.23	0.40	0.65
Ours	**0.09**	**0.23**	**0.42**	**1.25**	**0.09**	**0.14**	**0.25**	**0.48**	**0.10**	**0.15**	**0.26**	**0.47**	**0.12**	**0.24**	**0.39**	**0.67**	**0.11**	**0.21**	**0.38**	**0.94**	**0.08**	**0.18**	**0.33**	**0.61**

**Table 6:** Quantitative depth upsampling results (in MAD) on ToFMark dataset.

	Books	Devil	Shark
Bicubic	16.23	17.78	16.66
JGF [9]	17.39	19.02	18.17
TGV [37]	12.80	14.97	15.53
SRGAN [5]	11.76	12.80	13.92
ESRGAN [6]	10.44	12.16	13.03
DJIF [46]	10.85	11.63	13.50
DSR [26]	10.32	10.41	12.59
CGN [27]	10.01	10.23	11.87
Ours	**9.69**	**9.14**	**11.44**

**Table 7:** Quantitative depth upsampling results (average PSNR) on the MVD test sequences Doorflowers, PoznanStreet, and PoznanCarpark.

	Doorflowers	PoznanStreet	PoznanCarpark
Bicubic	38.12	45.09	35.15
JGF [9]	38.36	45.28	35.13
TGV [37]	38.42	45.50	35.18
SRGAN [5]	39.95	45.67	36.60
ESRGAN [6]	40.81	47.73	38.09
DJIF [46]	40.67	47.69	38.26
DSR [26]	41.38	48.33	39.21
CGN [27]	41.80	48.72	39.66
Ours	**41.95**	**49.11**	**39.84**

## Data Availability

Data will be made available on request.
